# Identification of Potential Driver Genes and Pathways Based on Transcriptomics Data in Alzheimer's Disease

**DOI:** 10.3389/fnagi.2022.752858

**Published:** 2022-03-18

**Authors:** Liang-Yong Xia, Lihong Tang, Hui Huang, Jie Luo

**Affiliations:** School of Biomedical Engineering, Shanghai Jiao Tong University, Shanghai, China

**Keywords:** Alzheimer's disease, transcriptomics, drug repurposing, deep learning, drug-target interaction

## Abstract

Alzheimer's disease (AD) is one of the most common neurodegenerative diseases. To identify AD-related genes from transcriptomics and help to develop new drugs to treat AD. In this study, firstly, we obtained differentially expressed genes (DEG)-enriched coexpression networks between AD and normal samples in multiple transcriptomics datasets by weighted gene co-expression network analysis (WGCNA). Then, a convergent genomic approach (CFG) integrating multiple AD-related evidence was used to prioritize potential genes from DEG-enriched modules. Subsequently, we identified candidate genes in the potential genes list. Lastly, we combined deepDTnet and SAveRUNNER to predict interaction among candidate genes, drug and AD. Experiments on five datasets show that the CFG score of *GJA1* is the highest among all potential driver genes of AD. Moreover, we found *GJA1* interacts with AD from target-drugs-diseases network prediction. Therefore, candidate gene *GJA1* is the most likely to be target of AD. In summary, identification of AD-related genes contributes to the understanding of AD pathophysiology and the development of new drugs.

## Introduction

Alzheimer's disease (AD) is one of the most common neurodegenerative diseases, accounting for the majority of dementia patients (Wood, 2018; Darby et al., 2019). AD is estimated to affect in 13.8 million individuals in the United States (US), with 7.0 million being aged 85 years or older by 2050 (Alzheimer's Association, 2018; Cummings et al., 2019). Currently, genetic factor are believed to be partially responsible for AD (Xu et al., 2018). Genome-wide association studies (GWAS) have also revealed that some single nucleotide polymorphisms (SNPs) contribute to AD disease onset (Hao et al., 2019; Andrews et al., 2020). These include common variants such as amyloid protein precursor (*APP*), presenilin-1 (*PSEN1*), presenilin-2 (*PSEN2*) and apolipoprotein E (*APOE*). *PSEN1, PSEN2* and *APP* genes are clear pathogenic genes of early-onset AD (Lanoiselée et al., 2017). *APOE*, as the only identified risk gene for late-onset AD, can increase the rate of cognitive decline (Wijsman et al., 2011). Different microRNAs (miRNAs) are also involved in the pathophysiology of AD (Femminella et al., 2015). For example, miRNA-377 promotes cell proliferation and inhibits cell apoptosis by regulating the expression level of cadherin 13 (CDH13), thus participating in the occurrence and development of AD (Liu et al., 2018). Long non-coding RNAs (lncRNAs) have been widely reported to be associated with a variety of physiological and pathological processes, such as AD. Brain cytoplasmic RNA is a kind of lncRNA, and the overexpression of brain cytoplasmic may lead to synaptic/dendritic degeneration in AD (Doxtater et al., 2020). Despite the fact that remarkable advances have been made in the understanding of the genetic basis of AD, there is no disease modifying therapy for AD. Identification of AD-related genes from transcriptomics becomes an attractive strategy for finding potential targets for drug therapy.

Gene expression profiling of transcriptomic datasets of AD and normal brain samples has identified potential genes and contributed to the search for potential targets (Patel et al., 2019). Correlation networks are often used to analyze gene expression data and gather biologically-relevant information from genes with similar co-expression patterns. At present, the two most commonly used gene co-expression network algorithms are SWItchMiner (SWIM) (Falcone et al., 2019) and Weighted Gene Correlation Network Analysis (WGCNA) (Nangraj et al., 2020; Ren et al., 2020). SWIM constructs an unweighted correlation network using local and global graph attributes to mine genes, known as switch genes, that have been shown to be associated with drastic changes in cell phenotypes, such as cancer development. WGCNA builds a correlation network that can be weighted or unweighted, and identifies related genes by measuring the centrality of a gene in the network. However, SWIM does not consider scale-free networks. The most notable characteristic of a scale-free network is the relative commonness of vertices with a degree that greatly exceeds the average. The highest-degree nodes are often referred to as "hubs" and are considered to have a specific purpose in their network. WGCNA is based solely on a scale-free network that is used to determine the relationships between genes, thereby enabling the identification of modules (clusters) of highly correlated genes, and the hub gene in each module. WGCNA is ideal for the identification of gene modules and key genes that contribute to phenotypic traits. Here, we used WGCNA to mine AD-specific modules from DEGs of AD and normal samples and identified candidate genes of from AD-specific modules.

Studying target-drug-disease network has contributed to the search for candidate genes of AD. In recent years, deep learning has been applied in biomedical and artificial intelligence fields, and many deep learning frameworks have been used to deal with the prediction problem of drug-target interaction (DTIs) (Xia et al., 2019). Öztürk et al. (2018) proposed a convolutional neural network (CNN)-based method based on using only sequence information and performing DTIs prediction on Davis and KIBA dataset. Rayhan et al. developed the FRnet-DTI, which is using autoenconder and CNN for feature extraction and classification, respectively (Chu et al., 2021). Zeng et al. (2020a) utilized cascade deep forest and arbitrary-order neighboring algorithms to predict DTIs. Zeng et al. (2020b) developed deepDTnet, a deep learning methodology for new target identification and drug repurposing in a heterogeneous network embedding 15 types of chemical, genomic, phenotypic, and cellular network profiles. Lots of works has been proposed for drug repurposing. Zeng et al. (2019) presented deepDR (deep learning-based drug repositioning), to systematically infer new drug-disease relationships for *in silico* drug repurposing. Fiscon et al. (2021) proposed SAveRUNNER, which predicts drug-disease associations by quantifying the interplay between the drug targets and the disease-specific proteins in the human interactome via a novel network-based similarity measure that prioritizes associations between drugs and diseases locating in the same network neighborhoods. Here, we combined deepDTnet and SAveRUNNER to predict interaction among candidate genes, drug and AD.

**Figure 1:**
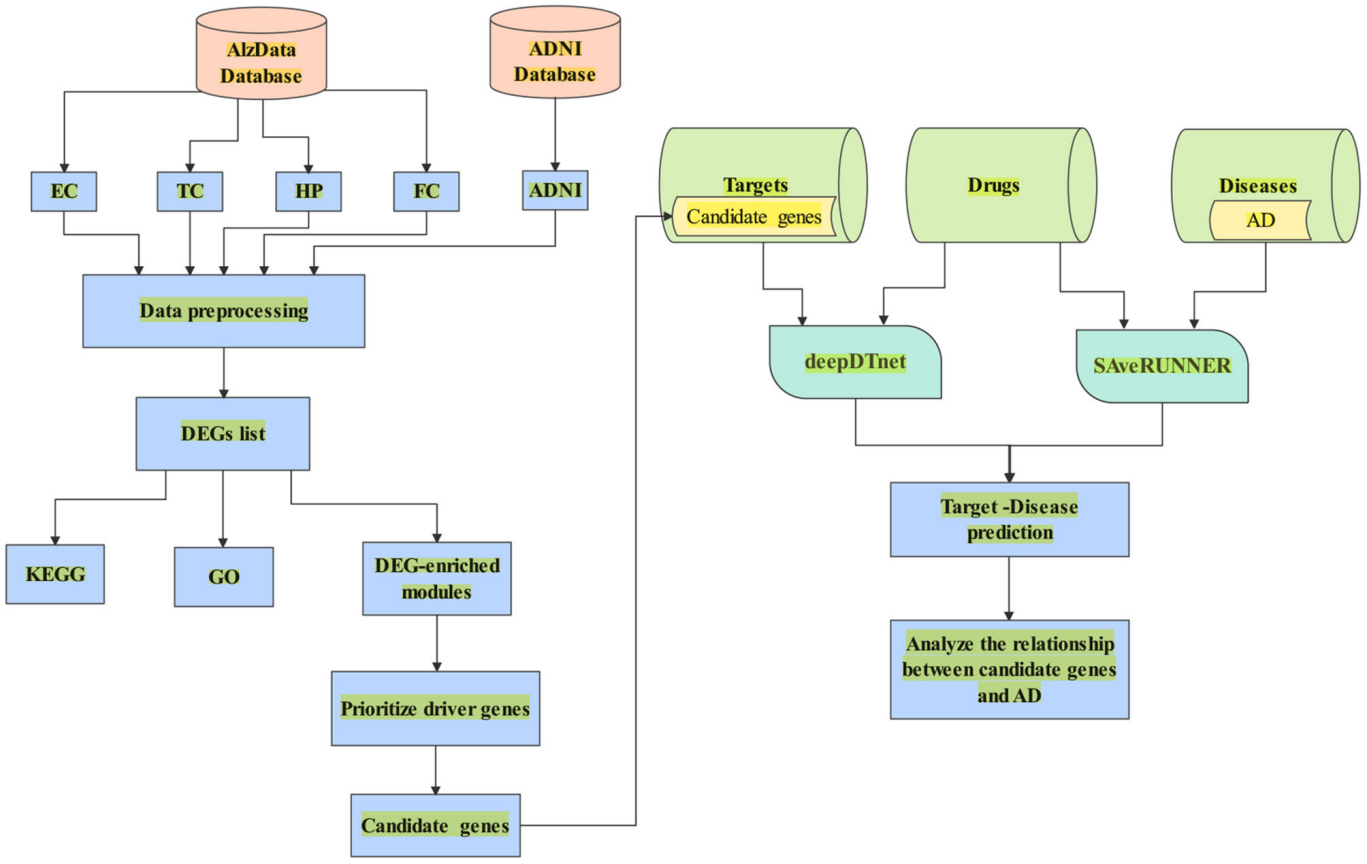
A flowchart of the whole study. (1) Data collection from AlzData and ADNI; (2) Data preprocessing (e.g., eliminating the samples with missing data); (3) DEGs regarded with |*logFC*| > 0.1 and *FDR* < 0.05; (4) Enrichment of biological process analyzed by DAVID 6.8; (5) Use WGCNA to find AD-specific module; (6) Prioritize driver genes of AD by CFG score; (7) candidate genes with *CFG*≥5 are identified. (8) Collect the dataset of target, drug and disease; (9) Combine deepDTnet and SAveRUNNER to predict association between candidate genes and AD.

In this paper, we aimed to search potential driver genes for AD from DEGs based on multiple transcriptomics dataset. We hypothesized that the DEGs might be regulated by several candidate genes in the DEG-enriched coexpression modules/networks by WGCNA. We used CFG score as a measurement of the likelihood for candidate genes to be AD targets. Further, we combined deepDTnet and SAveRUNNER to predict interaction between candidate genes and AD based on gene-drug-disease network in Figure 1.

## Materials and Methods

###  AD Expression Data Collection and Preprocessing

Our dataset came from the AlzData and ADNI database. For AlzData, Xu et al. constructed new database AlzData (http://www.alzdata.org/) including, hippocampus (HP), entorhinal cortex (EC), frontal cortex (FC), and temporal cortex (TC). The original four microarray data come from Gene Expression Omnibus (GEO) (https:// www.ncbi.nlm.nih.gov/geo), by searching with the keyword “Alzheimer.” Data retrieval has been performed using the following series of criteria: 1) AD-related expression profiles in the ArrayExpress database (https://www.ebi.ac.uk/arrayexpress/) were checked to avoid potential omissions; 2) Studies with no genome-wide probes or few probes were filtered; 3) For those GSE series with possibly duplicated samples or identical sample resource, we retained the one with a larger sample size and excluded another; 4) Only expression profiles of human postmortem brain tissues from HP, EC, FC, and TC, which were main regions affected by AD, were included; 5) Data retrieval and quality control were double-checked by two investigators. To ensure data quality, samples that were younger than 50 years old, or were outliers in our principal component analysis (PCA) of expression distribution, were excluded from this study.

For ADNI data (http://adni.loni.usc.edu), Gene expression profiling from peripheral blood samples collected using PAXgene tubes for RNA analysis was performed on the Affymetrix Human Genome U219 Array (www.affymetrix.com, Santa Clara, CA) for ADNI and on the Illumina Whole-Genome DASL assay (www.illumina.com, San Diego, CA) for AddNeuroMed and MCSA. All probe sets were mapped and annotated with reference to the human genome (hg19). Raw microarray expression values were pre-processed followed by standard quality control (QC) procedures on samples and probe sets. Briefly, raw expression values were pre-processed using the robust multi-chip average normalization method. We checked discrepancies between the reported sex and sex determined from sex-specific gene expression data including XIST and USP9Y and also evaluated whether SNP genotypes were matched with genotypes predicted from gene expression data.

In this study, we only consider gene expression data and binary classification problem (control vs. AD). After data processing, e.g., eliminating the samples with missing data, altogether, we have 467 controls and 309 AD from five dataset for subsequent analyses in total, including EC (39 vs. 39), HP (67 vs. 74), FC (128 vs. 104), TC (39 vs. 52) and ADNI (194 vs. 40). Detailed information of each dataset is shown in Table 1.

**Table 1:** Brief descriptions for five datasets.

**Dataset**	**AlzData**				**Alzheimer's disease neuroimaging initiative**
	**Entorhinal cortex**	**Hippocampus**	**Frontal cortex**	**Temporal cortex**	
Abbreviation	EC	HP	FC	TC	ADNI
No.of.gene	15361	16313	11779	15462	49387
Sample size(Control/AD)	78 (39/39)	141 (67/74)	232 (128/104)	91 (39/52)	234 (194/40)
Age	80 (29.6)	81.7 (9.6)	83 (9.4)	81 (8.7)	74.3 (6.5)
Male/Female/Unknown	35/43/0	68/73/0	99/111/22	32/41/18	116/118/0
*Aβ*	NA	NA	NA	NA	1142.9 (494.9)
Tau	NA	NA	NA	NA	25.4 (11.6)

*These datasets come from AlzData and ADNI, respectively. Each dataset has multiple features. SDs are given in parentheses*.

###  Statistical Analysis

Genes with log2 fold change greater than 0.1 (|*logFC*| > 0.1) and FDR smaller than 0.05 (*FDR* < 0.05) were defined as DEGs in AD patients in the each dataset. Functional enrichment of the DEGs was produced from Database for DAVID 6.8, which now provides a comprehensive set of functional annotation tools for investigators to understand biological meaning behind large list of genes. For obtained list of DEGs, DAVID 6.8 is able to identify enriched biological themes, particularly KEGG pathway and GO terms (Huang et al., 2007). Differential expression analysis was conducted by R package limma and the Benjamini-Hochberg's method was used to correct for multiple comparisons (Xu et al., 2018).

###  Weighted Gene Co-expression Network Analysis

We used R package WGCNA to perform the weighted correlation network analysis. For genes i and j, the correlation coefficient is *r*_*ij*_, we define the correlation intensity : $a_{ij}=r_{ij}^{\beta }$, which depends on the choice of power β (the power value ranging from 1 to 20). When the independence is more than 0.80, the scale-free network is obtained by screening the appropriate power value. Finally, the adjacency matrix was transformed into topological overlap matrix (TOM). Once the network is built through the TOM, it is converted to a distance matrix (1-TOM) to use it as the basis for clustering. A dynamic tree-cutting algorithm is then applied to the dendrogram to generate a partition of disjunct sets of genes. In addition, we extracted the corresponding gene information of each module for further analysis (Bot́ıa et al., 2017).

###  deepDTnet and SAveRUNNER

In this study, we combined deepDTnet and SAveRUNNER to predict interaction between candidate genes and AD. deepDTnet and SAveRUNNER were applied to predict the interactions of candidate genes/targets and drugs and relationship drugs and diseases, respectively.

Firstly, deepDTnet uses stacked denoising autoencoder (SDAE) to obtain low-dimensional embedding for both drugs and targets. A SDAE model minimizes the regularized problem and tackles reconstruction error, defined as follows:


(1) $$\begin{array}{r} min_{w_{l},b_{l}}||x-\hat{x}|{|}_{F}^{2}+\lambda \underset{l}{\sum }||W_{l}|{|}_{F}^{2} \end{array}$$

where x is input sample x(a vector); L is the number of layers, *w*_*l*_ is weight matrix, and *b*_*l*_ is bias vector of layer *l*∈{1, ., *L*}. λ is a regularization parameter and ||.||_*F*_ denotes the Frobenius norm. The middle layer is the key that enables SDAE to reduce dimensionality and extract effective representations of side information.

Subsequently, Positive Unlabeled-matrix completion is used to predict unknown drug-target pairs. Assume the drug-target interaction matrix is given as $P\in R^{N_{d}\times N_{t}}$, where *N*_*d*_ is the number of drugs and *N*_*t*_ is the number of targets. When *P*_*ij*_ = 1, infers drug i is linked to target j while zero indicates the relationship is unobserved. The optimization problem of our model is parameterized as:


(2) $$\begin{array}{r} \underset{i,j}{m}in\underset{\left(i,j\right)\in \Omega^{+}}{\sum }{\left(P_{ij}-x_{i}WH^{T}y_{j}^{T}\right)}^{2}+\alpha \underset{\left(i,j\right)\in \Omega^{-}}{\sum }{\left(P_{ij}-x_{i}WH^{T}y_{j}^{T}\right)}^{2}+\lambda \left(||W|{|}_{F}^{2}+||H|{|}_{F}^{2}\right) \end{array}$$

where the set Ω∈*N*_*d*_ × *N*_*t*_ is the observed entries from the true underlying matrix that includes both positive and negative entries, such that Ω = Ω^+^∪Ω^−^, let Ω^+^ denotes the observed samples and Ω^−^denotes the missing entries chosen as negatives. Under the assumption that the matrix is modeled to be low rank, i.e., *W*∈*N*_*d*_ × *k* and *H*∈*N*_*t*_ × *k*, and these matrices share a low dimensional latent space, satisfying *k* ≤ *N*_*d*_, *N*_*t*_. For biased inductive matrix completion, the value α is the key parameter, λ is a regularization parameter. Next, we approximate the likelihood of the pairwise interaction score between drug i and target j as:


(3) $$\begin{array}{r} Score\left(i,j\right)=x_{i}WH^{T}y_{j}^{T} \end{array}$$

where the higher score means a higher possibility that drug i is correlated with target j.

Then, to quantify the vicinity between drug and disease modules, SAveRUNNER implements a novel network similarity measure:


(4) $$\begin{array}{r} f\left(p\right)=\frac{1}{1+e^{-c\left[\frac{\left(1+QC\right)\left(m-p\right)}{m}d\right]}} \end{array}$$

Where p is the network proximity measure defined: $p\left(T,S\right)=\frac{1}{\left||T|\right|}\underset{t\in T}{\sum }mim_{s\in S}d\left(t,s\right)$ that represents the average shortest path length between drug targets t in the drug module T and the nearest disease genes s in the disease module S; QC is the quality cluster score; m is *max*(*p*); c and d are the steepness and the midpoint of *f*(*p*), respectively.

Finally, via deepDTnet and SAveRUNNER, we identified newly the relationship among candidate genes, drug and neurodegenerative diseases, which is including AD.

More detail about deepDTnet and SAveRUNNER could be found in previous study (Zeng et al., 2020b; Fiscon et al., 2021).

###  Convergent Functional Genomics

The potential driver genes was prioritized from AD-specific modules by CFG method, which integrated various levels of AD-related evidence (Ayalew et al., 2012; Xu et al., 2018). The range of CFG score was from 0 to 5, with 5 indicating highest priority. There were five AD-related evidence:1) Genetic association. If a gene had at least one locus being significantly associated with AD based on the summary statistics from the International Genomics of Alzheimer's Project [IGAP], 1 point was assigned; otherwise zero point. 2) Genetic regulation of gene expression. If a gene was associated with Expression Quantitative Trait Loci (eQTLs) showing an AD-risk in IGAP data, 1 point was assigned; otherwise zero point. 3) Protein-protein interaction. If a gene was physically interacted with any AD core genes (*APP, PSEN1, PSEN2, APOE*, or *MAPT*), 1 point was assigned; otherwise zero point. 4) Expression correlation with AD pathology. If the expression level of a gene was correlated with AD pathology in AD mice, 1 point was assigned; otherwise zero point. 5) Early alteration in AD mouse brain. If a gene showed differential expression in hippocampus of 2-month-old AD mice compared with age matched wild-type mice, 1 point was assigned; otherwise zero point.

## Results

###  DEG Detection

**Figure 2:**
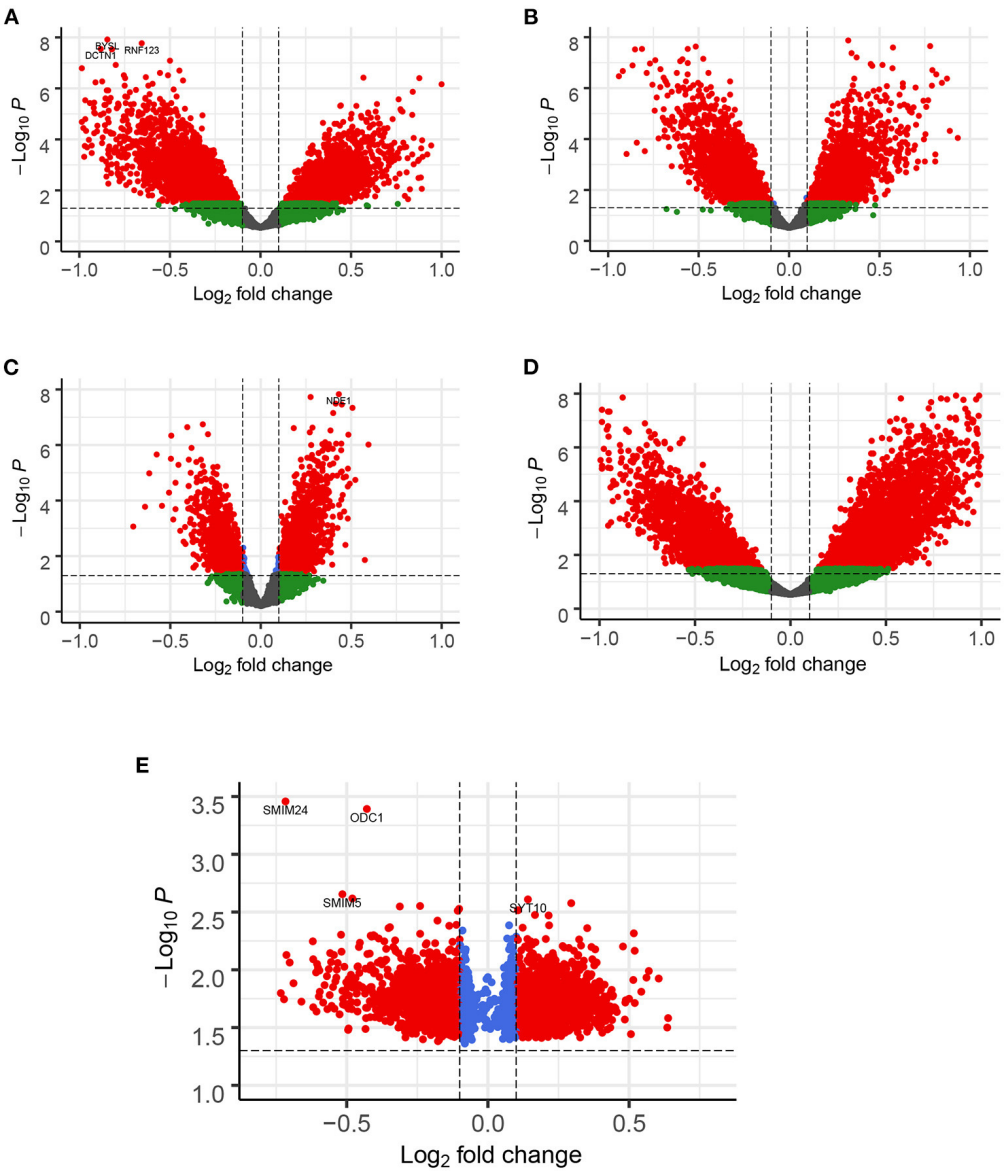
Enhanced Volcano for illustrating DEGs in all datasets. The gene with |*logFC*| > 0.1 and *FDR* < 0.05 as DEGs shown in red node. **(A)** EC, **(B)** HP, **(C)** FC, **(D)** TC and **(E)** ADNI. Note: in ADNI dataset, DEGs by counting the frequency of 3 or above out of 10 occurrences.

**Figure 3:**
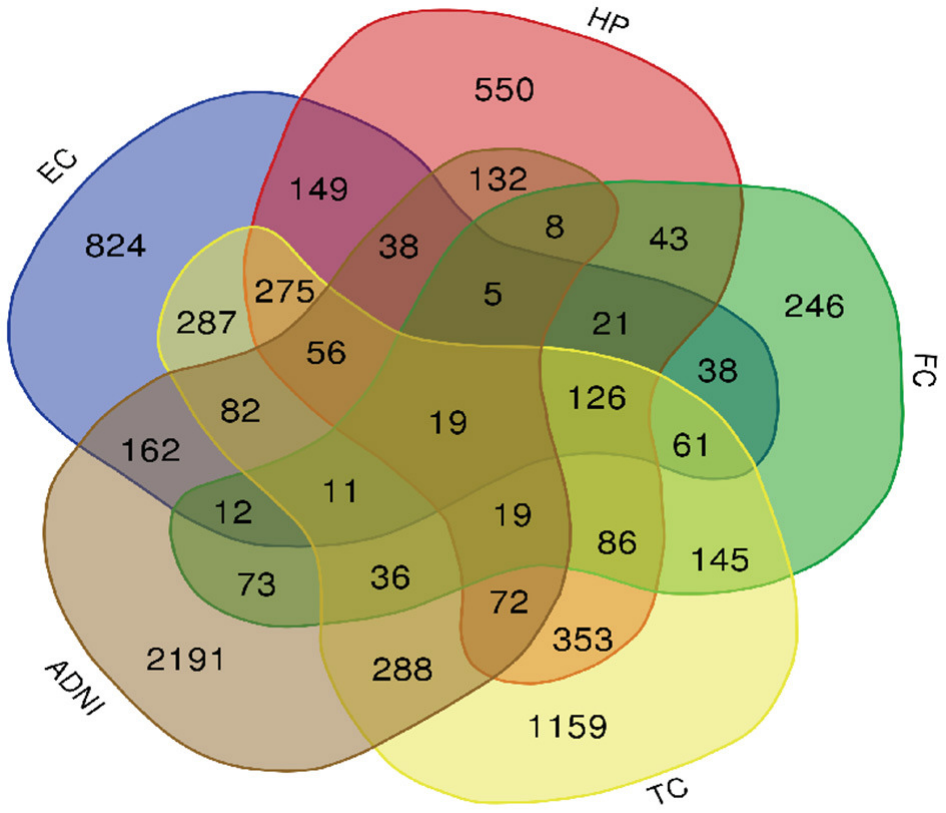
Venn diagram is used to represent relationships between EC (blue), HP (red), FC (green), TC (yellow) and ADNI (brown).

**Figure 4:**
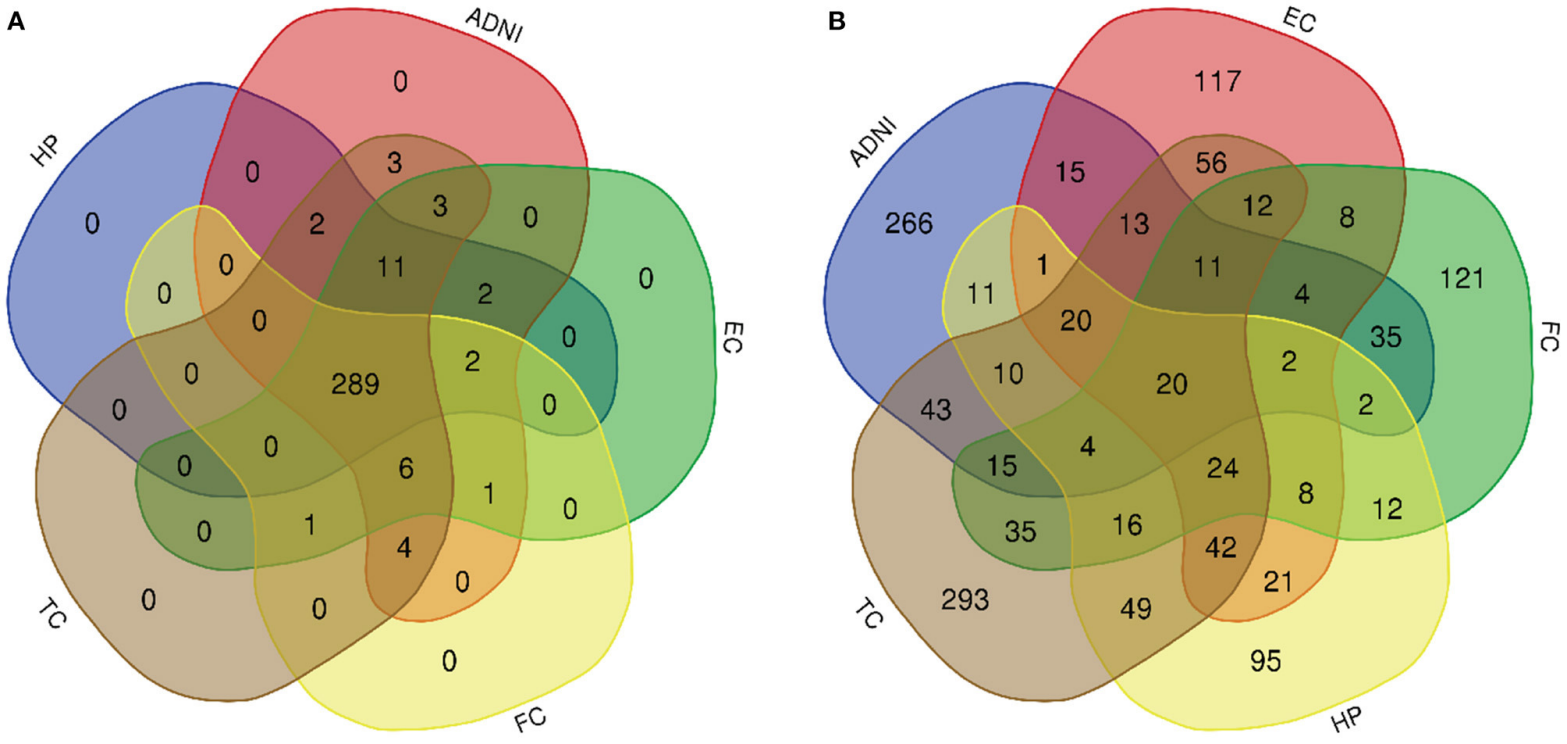
Venn diagram is used to represent relationships between multiple datasets. **(A)** KEGG pathway and **(B)** GO term.

**Figure 5:**
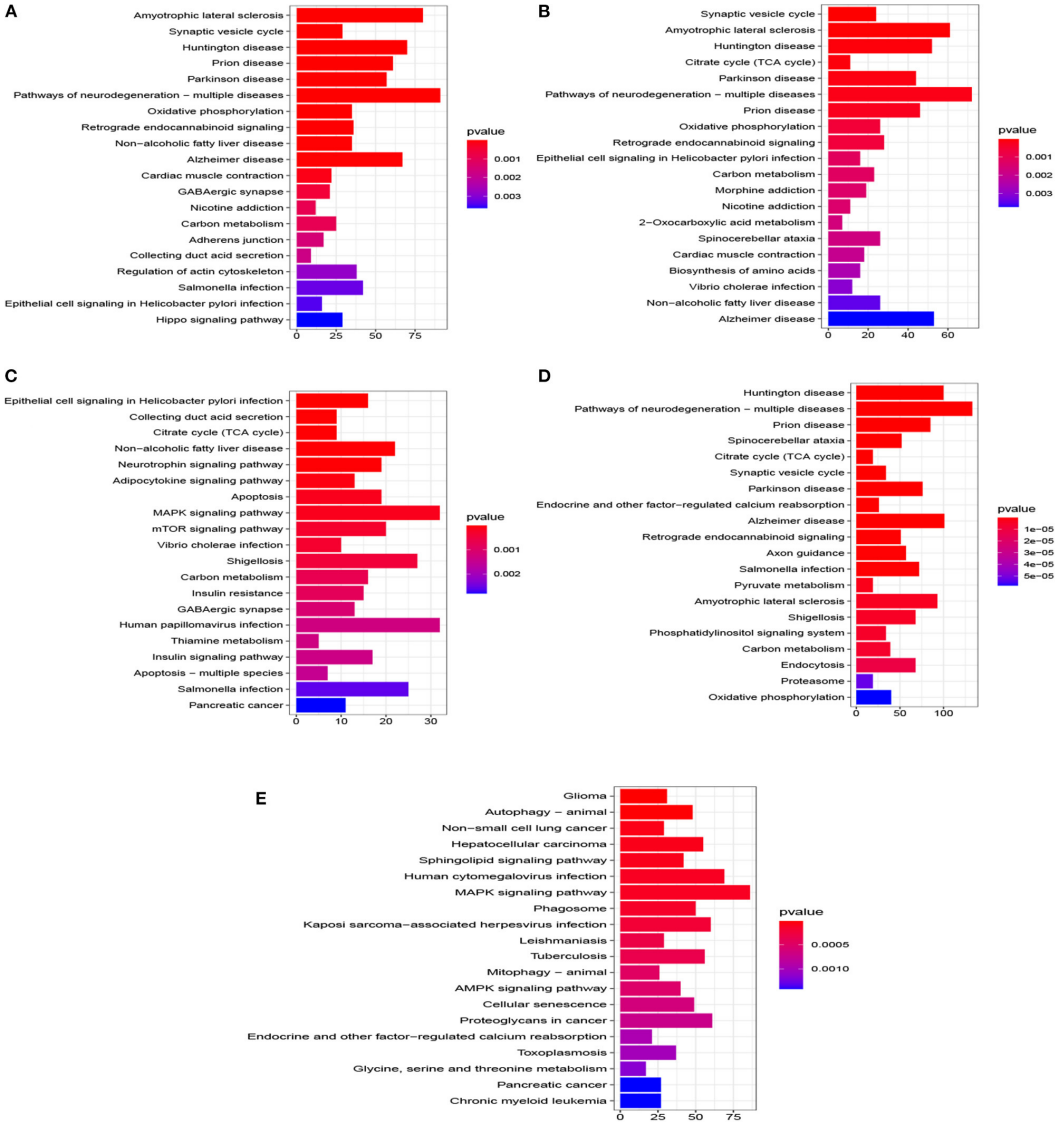
Top 20 pathway of KEGG for five datasets (*P* < 0.005). **(A)** EC, **(B)** HP, **(C)** FC, **(D)** TC, and **(E)** ADNI.

A total of 776 samples and 108,302 genes from multiple transcriptomic datasets were compiled for DEGs detection. Besides, for ADNI dataset, we randomly chose 40 samples from the control in 10 times and selected gene with frequency greater than or equal to 3. Each red node represented DEG for five datasets in Figure 2. We identified 7,567 DEG(2166 EC, 1952 HP, 949 FC, 3075 TC and 3204 ADNI) for subsequent analyses. About 6 19% of the total genes could be identified as DEGs. Among the DEG list in all five datasets, the expression patterns of well-known AD risk genes, such as APP, PSEN1, PSEN2, APOE and MAPT were only slightly altered or unchanged in AD patients. In addition, 19 genes had a consistently differential expression from EC, HP, FC, TC and ADNI (Figure 3). We investigated functional enrichment of the AD-related DEGs. The 7,567 target genes in the network were enriched in 324 KEGG pathway and 1,381 GO terms in Figure 4. We identified 61 KEGG pathway and 324 GO terms (*P*< *0.005*), respectively. As shown in Table 2, we also found several pathways have been reported to be associated with AD, including Alzheimer's disease pathway, *MAPK* signaling pathway and AMPK signaling pathway. Top 20 significantly KEGG pathway selected was exhibited for each dataset in Figure 5. Besides, these GO terms are divided into ontologies based on a hierarchical relations. Specifically, DEGs related to the biological processes for synaptic-related functions were significant enriched in Table 3, such as chemical synaptic transmission, regulation of postsynaptic membrane potential, synaptic vesicle exocytosis, synaptic transmission, GABAergic, regulation of synaptic transmission, glutamatergic, synaptic vesicle endocytosis and long-term synaptic potentiation. In addition, they were associated with neuron-related processes, including neurotransmitter secretion, neuron projection morphogenesis, negative regulation of neuron apoptotic process and negative regulation of neuron projection development.

**Table 2:** Significant KEGG pathways obtained from DAVID (*P* < 0.005).

**ID**	**Description**	**ID**	**Description**
hsa00020	Citrate cycle (TCA cycle)	hsa04966	Collecting duct acid secretion
hsa00190	Oxidative phosphorylation	hsa05010	Alzheimer's disease
hsa00260	Glycine, serine and threonine metabolism	hsa05012	Parkinson's disease
hsa00620	Pyruvate metabolism	hsa05014	Amyotrophic lateral sclerosis
hsa01200	Carbon metabolism	hsa05016	Huntington disease
hsa01210	2-Oxocarboxylic acid metabolism	hsa05017	Spinocerebellar ataxia
hsa01230	Biosynthesis of amino acids	hsa05020	Prion disease
hsa01522	Endocrine resistance	hsa05022	Pathways of neurodegeneration - multiple diseases
hsa03050	Proteasome	hsa05032	Morphine addiction
hsa04010	MAPK signaling pathway	hsa05033	Nicotine addiction
hsa04070	Phosphatidylinositol signaling system	hsa05110	Vibrio cholerae infection
hsa04071	Sphingolipid signaling pathway	hsa05120	Epithelial cell signaling in Helicobacter pylori infection
hsa04110	Cell cycle	hsa05131	Shigellosis
hsa04120	Ubiquitin mediated proteolysis	hsa05132	Salmonella infection
hsa04137	Mitophagy - animal	hsa05140	Leishmaniasis
hsa04140	Autophagy - animal	hsa05145	Toxoplasmosis
hsa04144	Endocytosis	hsa05152	Tuberculosis
hsa04145	Phagosome	hsa05163	Human cytomegalovirus infection
hsa04152	AMPK signaling pathway	hsa05167	Kaposi sarcoma-associated herpesvirus infection
hsa04211	Longevity regulating pathway	hsa05169	Epstein-Barr virus infection
hsa04218	Cellular senescence	hsa05202	Transcriptional misregulation in cancer
hsa04260	Cardiac muscle contraction	hsa05205	Proteoglycans in cancer
hsa04360	Axon guidance	hsa05212	Pancreatic cancer
hsa04625	C-type lectin receptor signaling pathway	hsa05214	Glioma
hsa04666	Fc gamma R-mediated phagocytosis	hsa05215	Prostate cancer
hsa04721	Synaptic vesicle cycle	hsa05219	Bladder cancer
hsa04722	Neurotrophin signaling pathway	hsa05220	Chronic myeloid leukemia
hsa04723	Retrograde endocannabinoid signaling	hsa05223	Non-small cell lung cancer
hsa04920	Adipocytokine signaling pathway	hsa05225	Hepatocellular carcinoma
hsa04932	Non-alcoholic fatty liver disease	hsa05235	PD-L1 expression and PD-1 checkpoint pathway in cancer
hsa04961	Endocrine and other factor-regulated calcium reabsorption		

**Table 3:** Significant GO terms obtained from DAVID (*P* < 0.005).

**ID**	**Term**
GO:0002223	Stimulatory C-type lectin receptor signaling pathway
GO:0006888	ER to Golgi vesicle-mediated transport
GO:0048015	Phosphatidylinositol-mediated signaling
GO:0038128	ERBB2 signaling pathway
GO:0007249	I-kappaB kinase/NF-kappaB signaling
GO:0006672	ceramide metabolic process
GO:0000165	MAPK cascade
GO:0045944	Positive regulation of transcription from RNA polymerase II promoter
GO:0007269	Neurotransmitter secretion
GO:0035329	Hippo signaling
GO:0006120	Mitochondrial electron transport, NADH to ubiquinone
GO:0042776	Mitochondrial ATP synthesis coupled proton transport
GO:0070125	Mitochondrial translational elongation
GO:0032981	Mitochondrial respiratory chain complex I assembly
GO:0007409	Axonogenesis
GO:0048812	Neuron projection morphogenesis
GO:0043524	Negative regulation of neuron apoptotic process
GO:0007268	Chemical synaptic transmission
GO:0060078	Regulation of postsynaptic membrane potential
GO:0016079	Synaptic vesicle exocytosis
GO:0048813	Dendrite morphogenesis
GO:0090263	Positive regulation of canonical Wnt signaling pathway
GO:0009967	Positive regulation of signal transduction
GO:0051932	Synaptic transmission, GABAergic
GO:0046034	ATP metabolic process
GO:0070933	Histone H4 deacetylation
GO:0007420	Brain development
GO:0007417	Central nervous system development
GO:0035357	Peroxisome proliferator activated receptor signaling pathway
GO:0015986	ATP synthesis coupled proton transport
GO:0040029	Regulation of gene expression, epigenetic
GO:0007399	Nervous system development
GO:0051966	Regulation of synaptic transmission, glutamatergic
GO:0048488	Synaptic vesicle endocytosis
GO:0010977	Negative regulation of neuron projection development
GO:0060071	Wnt signaling pathway, planar cell polarity pathway
GO:0006521	Regulation of cellular amino acid metabolic process
GO:2000310	Regulation of N-methyl-D-aspartate selective glutamate receptor activity
GO:0038061	NIK/NF-kappaB signaling
GO:0035418	Protein localization to synapse
GO:0060291	Long-term synaptic potentiation

*The first column is GO terms ID; the second column is the name of GO terms*.

**Figure 6:**
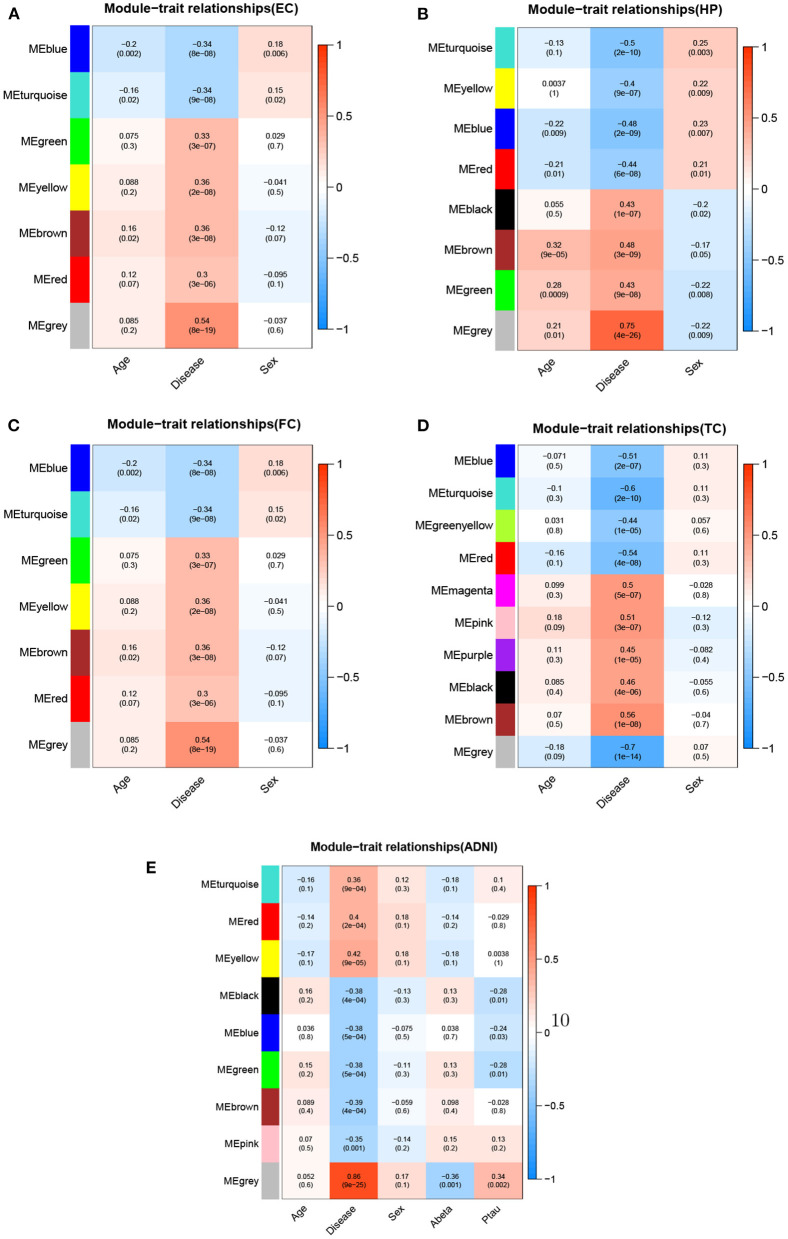
Module-trait relationships for five datasets.Each row represents different gene co-expression modules, and each column represents different clinical phenotypes. Number represent correlation coefficients and P-values are in parenthesis. Correlation strength is represented by continuous color, with red being positive, blue being negative. **(A)** EC, **(B)** HP, **(C)** FC, **(D)** TC, and **(E)** ADNI.

We used WGCNA to divide the DEGs into several highly related gene modules. As shown in Figure 6, a very significant positive correlation was observed between five modules and AD for five dataset. A modular size was ranged from 96 to 142 genes that might reflect the different layers and complexity of gene regulation in the AD brain. These five AD-specific modules were used for identifying potential driver genes for AD etiology and pathology. We obtained potential driver genes from each AD-specific modules for every dataset. Finally, after removing the overlap genes, we have 602 candidate genes from 5 AD-specific modules in total, including EC (107), HP(140), FC(142), TC(136) and ADNI(96). We hypothesized that the higher the CFG score is, the more likely the candidate genes are to be AD targets. We chose 40 genes with *CFG* ≥ 4 for subsequent analyses.

###  Identification and Prioritization of Potential Driver Genes

The 40 potential driver genes are prioritized by the CFG method based on AlzData database, which is integrated various levels of AD-related data in Table 4. For each gene, we showed the eQLT, GWAS, PPI, Early_DEG, Pathology correlation *Aβ* and Tau (*CFG* ≥ 4), and CFG score. We found that several genes were validated by previous studies from literatures. For example, *GJA1*, also known as connexin 43, shows upregulated mRNA and protein levels in AD (Ren et al., 2018). Specific reductions of RPH3A immunoreactivity compared with aged controls. *RPH3A* loss correlated with dementia severity, cholinergic deafferentation, and increased *Aβ* concentrations. Furthermore, *RPH3A* expression is selectively downregulated in cultured neurons treated with *Aβ* 25–35 peptides (Tan et al., 2014). *CASP6* activity is intimately associated with the pathologies that define AD, correlates well with lower cognitive performance in aged individuals, and is involved in axonal degeneration in several cellular and *in vivo* animal models (LeBlanc, 2013). The levels of angiotensinogen (*AGT*) is increased in the cerebrospinal fluid of patients with mild cognitive impairment and AD (Mateos et al., 2011). The stromal cell-derived factor 1 (SDF1), known as chemokine *CXCL12*, was a proinflammatory chemokine, highly expressed in the central nervous system. They may regulate synaptic transmission in excitability neurons and modulate neuroglial communication. *CXCL12* was detected in plasma and hippocampus AD patients. Levels of this chemokine were considerably decreased compared to the control group (Dulewicz et al., 2020). In summary, combining WGCNA with CFG offer a useful tool to prioritize potential genes for AD.

**Table 4:** The 40 potential driver genes are prioritized by the CFG method based on AlzData database.

***Gene***	**AD-related evidence**						***CFG***
	***eQTL***	***GWAS***	***PPI***	***Early_DEG***	***Pathology cor***		
					***(Aβ)***	***(Tau)***	
*GJA1*	2	2	*PSEN1, MAPT, APOE*	yes	0.388^**^	0.131^*ns*^	5
*FOXO1*	1	0	*PSEN2*	yes	0.270^*ns*^	0.526^*^	4
*PRKX*	3	NA	*PSEN1*	yes	0.352^*^	–0.023^*ns*^	4
*RPH3A*	5	2	*-*	yes	–0.199^*ns*^	–0.738^**^	4
*CASP6*	5	0	*APP, PSEN1, PSEN2, MAPT*	yes	0.482^***^	0.738^**^	4
*CRMP1*	1	3	*MAPT*	NA	–0.304^*^	–0.506^*ns*^	4
*RGS4*	1	32	*-*	yes	–0.419^**^	–0.579^*^	4
*NPTX2*	1	1	*-*	yes	–0.688^***^	–0.783^***^	4
*RPS27*	1	0	*PSEN2*	yes	0.503^***^	0.662^**^	4
*MEGF10*	3	8	*-*	yes	0.559^***^	0.120^*ns*^	4
*AP2A1*	1	0	*APP, PSEN2, MAPT*	yes	–0.277^*ns*^	–0.585^*^	4
*PITPNC1*	10	1	*-*	yes	–0.128^*ns*^	–0.638^*^	4
*AGT*	1	0	*APP, PSEN1, APOE*	yes	–0.359^*^	0.002^*ns*^	4
*AQP4*	7	4	*-*	yes	0.800^***^	0.275^*ns*^	4
*MYT1L*	3	12	*-*	yes	–0.488^***^	–0.583^*^	4
*IQGAP1*	1	0	*PSEN1*	yes	0.310^*^	0.282^*ns*^	4
*IGFBP7*	8	0	*MAPT, APOE*	yes	0.353^*^	0.510^*ns*^	4
*CITED2*	1	0	*APP, PSEN1, APOE*	yes	–0.433^**^	–0.772^***^	4
*SMAD1*	16	1	*APP, APOE*	NA	–0.332^*^	–0.497^*ns*^	4
*CDH7*	0	1	*PSEN1*	yes	–0.345^*^	–0.691^**^	4
*MSRB2*	5	2	*-*	yes	0.32^*^	0.609^*^	4
*DBI*	1	1	*-*	yes	0.780^***^	0.718^**^	4
*PELI2*	2	0	*PSEN2*	yes	0.591^***^	–0.107^*ns*^	4
*AVEN*	1	1	*-*	yes	0.525^***^	0.008^*ns*^	4
*F13A1*	7	3	*APP, APOE*	NA	0.195^*ns*^	0.623^*^	4
*SLA*	1	0	*PSEN1, MAPT*	yes	0.114^*ns*^	0.662^**^	4
*ADAMTS20*	2	17	*-*	yes	0.085^*ns*^	0.587^*^	4
*RARB*	6	2	*PSEN2*	yes	–0.064^*ns*^	–0.387^*ns*^	4
*SDC2*	8	3	*PSEN1, PSEN2, MAPT, APOE*	yes	0.041^*ns*^	0.086^*ns*^	4
*DCN*	8	0	*APP, PSEN1, MAPT, APOE*	yes	–0.416^**^	0.546^*^	4
*CCR5*	1	0	*APP*	yes	0.769^***^	0.616^*^	4
*GPRC5B*	2	41	*-*	yes	0.307^*^	–0.248^*ns*^	4
*IRF5*	1	0	*APP, PSEN1, PSEN2, MAPT, APOE*	yes	0.879^***^	0.839^***^	4
*IGFBP7*	8	0	*MAPT, APOE*	yes	0.353^*^	0.510^*ns*^	4
*CXCL12*	1	0	*APP, PSEN2, MAPT, APOE*	yes	0.432^**^	–0.069^*ns*^	4
*CREM*	1	0	*PSEN1, MAPT, APOE*	yes	–0.439^**^	–0.396^*ns*^	4
*EHHADH*	14	0	*MAPT, APOE*	yes	0.438^**^	–0.022^*ns*^	4
*SLC1A3*	7	1	*-*	yes	0.651^***^	0.494^*ns*^	4
*VAV3*	0	5	*MAPT*	yes	0.319^*^	–0.284^*ns*^	4
*IL15*	2	18	*-*	yes	0.623^***^	0.685^**^	4

“NA,” not applicable due to missing related data for the target gene. AD, Alzheimer's disease; CFG, convergent functional genomics score based on the total number of lines of AD-related evidence; DEG, differentially expressed gene; eQTL, the total number of risk SNPs based on the IGAP data setthat were able to regulate expression of the target gene; GWAS, the total number of risk SNPs within the target gene based on the IGAP data set; PPI, AD core genes (APP, PSEN1, PSEN2, MAPT, and APOE) that had a significant protein-protein interaction with the target genes; Early_DEG: target gene is differentially expressed in AD mouse models before AD pathology emergence; Expression correlation of the target gene and AD pathology in AD mice was performed for the Aβ line AD mice in Mouse (marked as Aβ) and the Tau line AD mice in Mouse (marked as Tau). *P < 0.05; **P < 0.01; ^***^P < 0.001.

###  Candidate Genes *GJA1*

**Figure 7:**
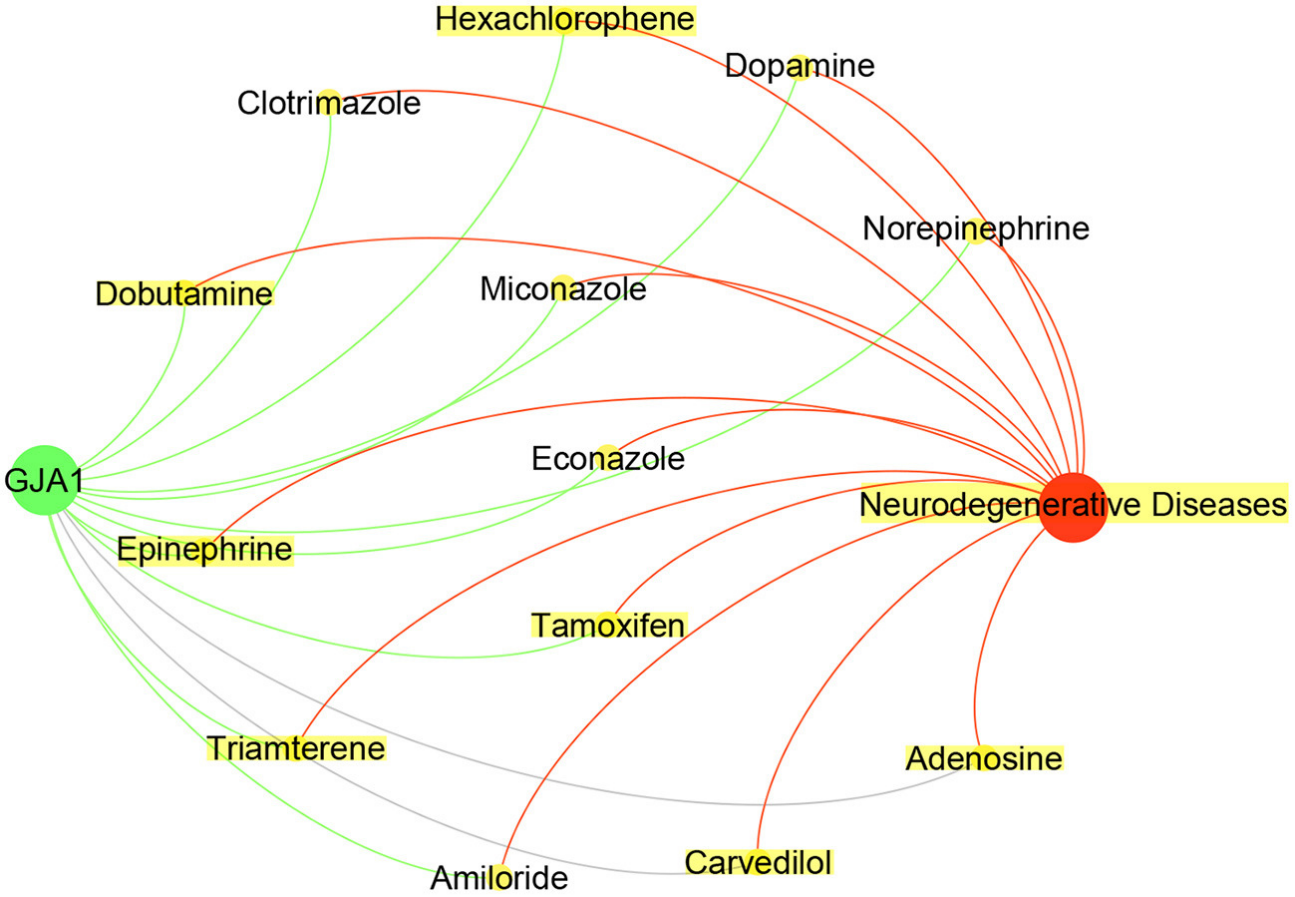
Drug-GJA1-disease interaction network. The network contained candidate target GJA1 (green), Neurodegenerative Diseases (red) and 13 drugs (yellow).Gray indicate known interaction. Green and red lines and newly predicted interactions using deepDTnet and SAveRUNNER, respectively.

As shown in Table 4, the CFG score of *GJA1* is the highest among all potential genes and regarded as candidate gene. We combined deepDTnet and SAveRUNNER to search association between candidate genes *GJA1* and AD based on target-drug-disease network. As shown in Figure 7, the network is constructed 13 drugs, a candidate genes *GJA1* and neurodegenerative diseases. 11 newly drug-target interaction and 13 newly drug-disease association are identified by deepDTnet and SAveRUNNER, respectively. Especially, we found that dopamine were validated by previous studies from literatures. Dopamine, a compound of the catecholamine and phenethylamine families playing important roles in the human brain, was predicted by deepDR to be associated with AD. Such a prediction can be supported by a previous study indicating that lack of dopamine in the brain may cause some of the earliest symptoms of Alzheimer (Zeng et al., 2019). In AD, the dysfunction of dopaminergic transmission has been hypothesized as a new player in the pathophysiology of AD. Dopamine acts through five different types of receptors, generally distinct in two main subclasses: D1-like [comprising the dopamine 1 receptor (D1R) and the dopamine 5 receptor (D5R)]; and D2-like [comprising the dopamine 2 receptor (D2R), dopamine 3 receptor (D3R) and the dopamine 4 receptor (D4R)]. Pan et al. found that dopamine, D1R and D2R concentration levels were decreased in patients with AD compared with controls. Moreover, decreased levels of dopamine and D2-like receptors were linked with the pathophysiology of AD because of their strong higher rank correlations with AD (Pan et al., 2020). To conclude, candidate genes *GJA1* is the most likely to be targets of AD.

## Discussion

Pathway enrichment analysis was performed to interpret the function of these DEGs. KEGG pathway analysis for the 7,567 DEGs were significantly enriched in one KEGG pathway “*MAPK* signaling pathway,” which is composed of ERK, P38, and JNK. In the adult nervous system, ERK activation is necessary for synaptic plasticity and memory formation (Du et al., 2019). In the brains of AD patients, P38 is highly expressed. *Aβ*-induced P38 activation increases tau phosphorylation and promotes the amyloidogenic processing of APP (Giraldo et al., 2014; Gourmaud et al., 2015). In a mouse model of AD, the JNK signaling pathway is overactivated in the spine before cognitive decline (Sclip et al., 2014). These studies indicate that the overactivation of MAPK signaling pathway could cause the occurrence of AD. Therefore, preventing MAPK overactivation is effective strategy in order to reduce *Aβ* deposition, Tau hyperphosphorylation, neuronal apoptosis, and memory impairment. MAPKs could be potential targets for novel and effective therapeutics of AD (Yenki et al., 2013; Feld et al., 2014).

GO term analysis indicated that the 7,567 DEGs were mainly involved in chemical synaptic transmission, regulation of postsynaptic membrane potential, synaptic vesicle exocytosis, synaptic transmission, GABAergic synapses, regulation of synaptic transmission, glutamatergic, synaptic vesicle endocytosis, long-term synaptic potentiation, neurotransmitter secretion, neuron projection morphogenesis, negative regulation of neuron apoptotic process and negative regulation of neuron projection development. Damage to neuronal and synaptic function has always been considered an important pathological feature of neurodegenerative diseases, and decreased synaptic activity is also considered to be the most relevant pathological feature of AD cognitive impairment (Wu et al., 2019). For example, the downregulation of GABAergic synapses is closely related to the loss of GABAergic inhibition (Kim et al., 2020). Studies have found that GABAergic neurotransmission is closely related to various aspects of AD pathology, including *Aβ* toxicity and Tau hyperphosphorylation (Kadoyama et al., 2021). The level of GABA inhibitory neurotransmitter in AD patients was significantly reduced, suggesting that AD has insufficient synaptic function and neuronal transmission (Schmitz et al., 2017). In addition, In a mouse model of AD indicate that the impairment of hippocampal neurogenesis may be mediated by GABAergic signal dysfunction or the imbalance between excitatory and inhibitory synapses (Sun et al., 2009). Therefore, GABAergic synapses not only plays an important role in the function of the hippocampus, but also in the pathogenesis of AD.

## Limitations

There are some limitations in this study. First, although we identified 23 potential driver genes of AD by the WGCNA and CFG method, these approachs could be used to prioritize genes rather than to identify true causal genes. Therefore, further biological validation of the identified genes are necessary in future studies. Second, 4 of 5 datasets were downloaded from AlzData, which only retained the common genes from different studies during the cross-platform normalization. Third, the sample size of EC, HP and TC available for analyze was still limited, and the larger sample size of FC and ADNI might have a greater influence on the results. Fourth, the rapid development of various omics provide new opportunities for understanding of AD. However, we only used transcriptomics dataset to identify potential driver genes of AD. Finally, more potential genes of AD were not considered. Deep learning has capacity to dig out more hidden gene in data and is a machine learning algorithm based on artificial neural network, which is a computational model inspired by the structure of human brain. The main difference between deep learning and traditional artificial neural network lies in the scale and complexity of network structure. The networks of deep learning have a larger number of hidden layers, while traditional artificial neural networks usually have only one hidden layer. This is due to the lack of big data and GPU hardware technical support in the last century. Due to the emergence of more powerful CPU and GPU hardware, deep learning with more hidden layers is proposed on the basis of artificial neural network, and more nodes can be used in each hidden layer (Esteva et al., 2019; Zou et al., 2019).

## Conclusions

In this study, we identified potential driver genes from AD-specific modules using multiple transcriptomics datasets and observed that DEGs were enriched with several pathways significantly by DAVID 6.8, which are consistent with observations from previous studies. Moreover, through studying of WGCNA, CFG and drug-target-disease network prediction, candidate gene GJA1 is the most likely to be targets of AD, actually reported in previous study. In summary, identification of AD-related genes contributes to the understanding of AD pathophysiology and the development of new drugs. In summary, Our results contribute to understanding pathophysiology of AD and looking for candidates drug targets.

## Data Availability Statement

The original contributions presented in the study are publicly available. This data can be found here: https://github.com/Macau-LYXia/Transcriptomics-Data-for-AD. Data used in the preparation of this article were obtained from the AlzData (http://www.alzdata.org/) and Alzheimer's Disease Neuroimaging Initiative (ADNI) database (adni.loni.usc.edu).

## Author Contributions

L-YX and LT contributed to collect data sets and analyze data. L-YX, LT, HH, and JL contributed to the interpretation of the results and revised the manuscript. L-YX took the lead in writing the manuscript. All authors contributed to the article and approved the submitted version.

## Funding

This work was supported by China Postdoctoral Science Foundation (2020M671125) and start-up grant of the Shanghai Jiao Tong University (WF220408213).

## Conflict of Interest

The authors declare that the research was conducted in the absence of any commercial or financial relationships that could be construed as a potential conflict of interest.

## Publisher's Note

All claims expressed in this article are solely those of the authors and do not necessarily represent those of their affiliated organizations, or those of the publisher, the editors and the reviewers. Any product that may be evaluated in this article, or claim that may be made by its manufacturer, is not guaranteed or endorsed by the publisher.
